# Understanding the Role of Triple Phase Boundaries on Coating-Free Solid-State Cathodes

**DOI:** 10.1021/acsenergylett.5c02607

**Published:** 2026-01-30

**Authors:** Longlong Wang, Bingkun Hu, Christopher Doerrer, Shengming Zhang, Lechen Yang, Liquan Pi, Max Jenkins, Boyang Liu, Shengda D. Pu, Yi Yuan, Hui Gao, Alex W. Robertson, Patrick S. Grant, Xiangwen Gao, Peter G. Bruce

**Affiliations:** † Future Battery Research Centre, Global Institute of Future Technology, 12474 Shanghai Jiao Tong University, Shanghai 200240, China; ‡ Department of Materials, University of Oxford, Oxford OX1 3PH, United Kingdom; § Department of Physics, 2707 University of Warwick, Coventry CV4 7AL, United Kingdom; ∥ Department of Chemistry, University of Oxford, Oxford OX1 3QZ, United Kingdom

## Abstract

Sulfide solid electrolytes have high ionic conductivities necessary to achieve high-rate solid-state cathodes at room temperature and low pressure. Cathode active materials generally require coatings to avoid deleterious oxidative decomposition reactions with the electrolyte. Coatings add cost and complexity to the manufacture. Here we decouple the effect of double and triple phase boundaries on the decomposition in the thick (i.e., ∼110 μm) uncoated solid state cathode. We show that more severe oxidative decomposition of solid electrolytes occurs when the cathode active materials, carbon, and the solid electrolyte coexist, highlighting the importance of the triple phase boundary concerning the decomposition. By regulating the electronic pathways at the triple phase boundary, a thick uncoated electrode at 1 mA cm^–2^ and 2 MPa stack pressure, delivers an initial areal capacity of ∼4.6 mAh cm^–2^ at 30 °C and ∼85% capacity retention after 500 cycles.

## Graphical abstract

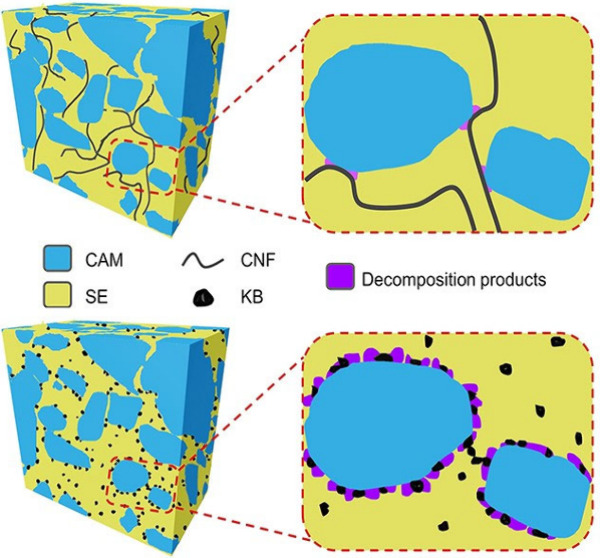

All-solid-state batteries (ASSBs) are viewed as a promising next-generation energy storage technology because of their inherent safety and potentially higher energy/power density compared with conventional liquid Li-ion batteries (LIBs).^1–3^ If ASSBs based on ceramic electrolytes are to deliver the high energy densities promised by such batteries, they must employ a high energy density anode, e.g., lithium metal, and a high energy density solid-state cathode (SSC) composed of the cathode active material (CAM) and a solid electrolyte (SE), as well as carbon to facilitate electronic transport through the SSC.^4–6^ Moreover, from the practical engineering point of view, the desired stack pressure should be ideally <2 MPa.^1,4,7–10^

Although sulfide-based solid electrolytes (SSEs) have the conductivities necessary to deliver high energy densities at practical rates (mA cm^–2^) and stack pressures less than a few MPa,^8,11–13^ they are readily oxidized at the potentials of nickel-rich CAMs, resulting generally in significant capacity loss on cycling.^14–19^ As a result, the CAM particles have to be coated with e.g. LiNbO_3_, Li_2_ZrO_3_ or LiZr_2_(PO_4_)_3_ to mitigate the reactivity.^8,12,20–24^ However, coatings add complexity and cost to the manufacture of batteries and can add a kinetic barrier to operation of the cell.^25–27^ It is desirable to avoid coatings if possible.^25,28^ Previous reports of uncoated CAM-based ASSBs have indicated that employing a low specific surface area carbon in the SSC could improve performance compared with using a large specific surface area carbon.^16,29–35^ The present study provides a mechanistic understanding, including the differences between decomposition products at the triple and double phase boundaries and its relationship to good performance under practical conditions.

Here we investigate the effect of double and triple phase boundaries on the SSE decomposition in the practical thick (i.e., ∼110 μm) SSC with a high CAM ratio (i.e., 75%) at practical cycling conditions (i.e., 1 mA cm^–2^ cycling rate and 2 MPa stack pressure). The SSCs comprise uncoated nickel-rich LiNi_0.83_Mn_0.06_Co_0.11_O_2_ (NMC), Li_6_PS_5_Cl, and either carbon nanofibers (CNFs) or Ketjen black (KB). We show that more severe oxidative decomposition of SSEs occurs when the CAM, carbon, and the SSE coexist compared with when only two phases (CAM/SSE or carbon/SSE) are present under practical conditions, highlighting the importance of the triple phase boundary. By regulating the electronic pathways at the triple phase boundary, a ∼110 μm thick uncoated electrode with CNFs at 1 mA cm^–2^ and 2 MPa stack pressure, delivers an areal capacity of ∼4.6 mAh cm^–2^ (182 mAh g^–1^ CAM utilization) at 30 °C on the first cycle (2.5 to 4.3 V) and ∼85% capacity retention after 500 cycles with a 0.03% capacity loss per cycle. Our findings provide new insights into the design of high-performance practical SSCs for ASSBs.

Figure 1 compares the load curves, capacity retention, and cycling efficiency of SSCs composed of CAMs, Li_6_PS_5_Cl and CNFs or KB. The diffraction patterns of the components are shown in Figure S1 and the morphologies are shown in Figure S2, with average particle sizes of several hundred nanometers for Li_6_PS_5_Cl, ∼20 nm for KB, and 1–5 μm for the single-crystal NMC particles. The CNF is graphitized carbon with a diameter of 50–200 nm and a length of 20–200 μm. The CAM loading is ∼ 25 mg cm^–2^ to ensure a high areal capacity and the cathode has a total thickness of ∼110 μm, as confirmed by the cross-sectional plasma focused ion beam scanning electron microscopy (PFIB-SEM) image in Figure S3. The corresponding morphology and microstructure of the SSCs with CNFs and KB are further revealed via PFIB-SEM 3D reconstruction and surface SEM analyses (Figure S4 and Figure S5). The same volumetric ratios of CAM: SE: carbon of 49:42:5 were used for CNFs and KB (Figure S4). The volumetric ratios were shown previously to give the highest capacity when cycled at 30 °C, 1 mA cm^–2^ rate and low stack pressures.^7,8,11–13^ A three-electrode cell with a lithium reference electrode measuring the SSC potential was used. A zero strain (no volume change) Li_4_Ti_5_O_12_ (LTO) based composite electrode, as shown in the SEM image (Figure S5), was used as the anode in this work to enable investigation of the SSC at higher current densities without any limiting effects of a Li metal anode such as the formation of lithium dendrites. The stack pressure was 2 MPa. On constant current cycling at 1 mA cm^–2^, the CNF-based SSC delivers an areal capacity of ∼4.6 mAh cm^–2^ at 30 °C (Figure 1a and Figure S6) and ∼5 mAh cm^–2^ at 60 °C (Figure S7), between 2.5 and 4.3 V, corresponding to CAM utilization of 182 mAh g^–1^ and 197 mAh g^–1^, respectively. The cycling efficiency of the first cycle is 80% and 84%, respectively, quickly reaching and remaining above 99.5% on continuous cycling (Figure 1c and Figure S7). The relatively low first-cycle Coulombic efficiency (CE) arises from the initial interfacial side reactions at the triple-phase boundaries (TPBs) and partial contact loss between the components. As cycling proceeds, both effects are mitigated: decomposition products form a passivating layer and contact loss reaches equilibrium, leading to the rapid increase in CE. Overall, despite the use of uncoated CAMs, it exhibits a capacity retention of 85% after 500 cycles with a 0.03% capacity loss per cycle, which is ∼12 times lower than KB-based SSCs (0.35% capacity loss per cycle, Figure 1b,d). Besides regulating the electronic pathways at the triple phase boundary, the improved performance at 2 MPa external stack pressure is also attributed to the zero-strain anode materials and single-crystal CAMs in this work.^7,10,11,36–46^

**Figure 1:**
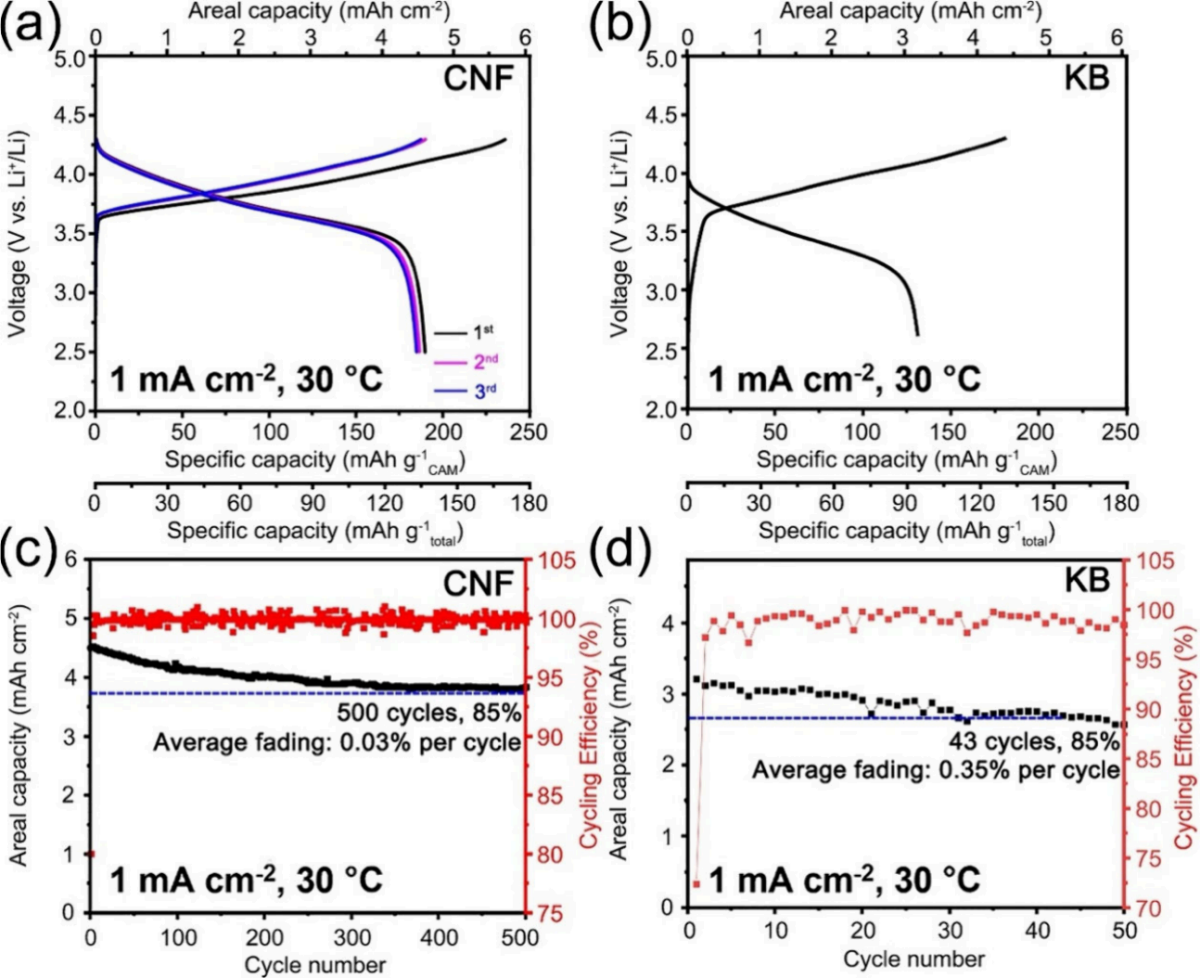
Electrochemical performance of the CNF- and KB-based solid-state cathodes with the same volumetric ratios of CAM: SE: carbon of 49:42:5 at 1 mA cm^–2^, 2 MPa stack pressure, and 30 °C. (a, b) The load curves of the CNF- and KB-based solid-state cathodes over the first few cycles. The specific capacity is calculated based on the mass of the cathode active material or the total mass of the solid-state cathode. (c, d) The areal capacity and cycling efficiency of the CNF- and KB-based solid-state cathodes.

In this work, we used LTO to avoid complications related to dendrite formation and focus on the cathode–electrolyte interfacial processes. Importantly, changing the anode does not alter the fundamental electrochemical evolution of the composite cathode. Therefore, the TPB regulation demonstrated here is expected to be applicable to lithium metal solid-state batteries. If the problems facing the realization of a Li metal anode were solved, as shown in Figure S8, a solid-state cell incorporating this cathode would deliver an energy density of 1115 Wh L^–1^ and 1210 Wh L^–1^ with a 20 μm thick Li anode and Li_6_PS_5_Cl separator (Table S1), at 30 and 60 °C, respectively, where it is compared with a LIB of today.^47–51^ Our calculation takes into account only the electrochemical cell components and not components, such as current collectors. The capacity retention and cycling efficiency for the CNF-based SSCs at 30 °C are comparable to those with coated CAM particles, but without of course the complexity of coatings.^8,12,20–24^ They are also starkly better than that with KB in the absence of coatings as Li_6_PS_5_Cl oxidizes above 2.1 V.^14–19^ From a manufacturing perspective, KB disperses readily and is widely used, but its very high surface area increases the demand on binder and solvent as well as slurry viscosity, which can lower tap/areal density and complicate calendaring. In contrast, CNFs can achieve high electronic percolation at lower loadings and retain fast conductive pathways after calendaring, but their fibrous morphology requires more careful dispersion (e.g., higher shear or longer mixing) to prevent agglomeration or anisotropy. These trade-offs suggest that CNFs enable reduced total carbon content while maintaining high conductivity, whereas KB offers simpler mixing at the expense of higher carbon/binder fractions.

The measured electronic conductivity of CNF-based SSCs is higher than that of SSCs containing KB (Figure S9). The improved electronic conduction of CNF-based SSCs arises from both the intrinsically higher electronic conductivity of CNFs (∼10^2^–10^3^ S cm^**–**1^ of CNFs vs ∼10–10^2^ S cm^**–**1^ of KB) and their fibrous morphology enabling long-range electronic transport.^33,52,53^ Moreover, the lower specific surface area (∼24 m^2^ g^–1^, Table S2) of CNFs results in fewer TPBs and hence fewer side reactions. In contrast, the nanoparticulate morphology and high surface area (∼1010 m^2^ g^–1^) of KB lead to short-range electronic pathways and a significantly higher density of TPBs, thereby promoting interfacial decomposition. These results demonstrate that CNFs enable efficient electronic percolation while minimizing undesirable side reactions, highlighting their dual role in optimizing electronic pathways and stabilizing TPBs.^16,29–33^ As for the surface chemistry, Kundu et al.^54^ and Kim et al.^55^ have reported that the oxygen-containing functional groups can affect decomposition reactions at the TPBs. As a result, all of the carbon additives here were treated under Ar/H_2_ at 500 °C and further dried at 300 °C under vacuum for 24h. The Fourier-transform infrared (FTIR) spectra (Figure S10) indicate no obvious difference among the four dried carbon additives including CNFs, KB, Super P (SP) and carbon nanotubes (CNTs).

Figure 2 shows results for SSCs composed of Li_6_PS_5_Cl, MnO_2_ and KB or CNFs, respectively. The electronic conductivities of high-nickel single crystal LiNi_*x*_Mn_*z*_Co_*y*_O_2_ (*x* + *y* + *z* = 1)^56–59^ and MnO_2_ ^60–62^ are broadly similar at ∼10^–3^–10^–5^ and ∼10^–4^–10^–6^ S cm^–1^, respectively, and our measurement confirm that SSCs with NMC or MnO_2_ have comparable electronic conductivities (Figure S11). Thus, replacing the active NMC with electronically inert MnO_2_ does not compromise the electronic conductivity. The component ratios are identical to NMC-based SSCs (volumetric ratios 49:42:5), therefore the effect of the different carbons can be seen. The higher specific surface area of KB results in greater SE oxidation. For MnO_2_-based SSCs, this is the only source of capacity. In contrast, with NMC present, there is deintercalation and now the greater direct oxidation of SE by KB compared with CNFs results in more decomposition products that increase the voltage polarization, reaching the voltage cutoff earlier (after the passage of less capacity).^16,30^ In addition to CNFs and KB, we further studied the influence of conductive additives, SP (∼62 m^2^ g^**–**1^) and CNTs (∼280–350 m^2^ g^**–**1^) by comparing the first charge profiles of solid-state cathodes (SSCs) with NMC or MnO_2_ (Figure S12).^35^ SP, as a carbon black with a relatively low specific surface area (though higher than that of CNFs), provides efficient electronic conduction but introduces more TPBs, thereby promoting greater SE decomposition than CNFs. CNTs, with their one-dimensional morphology and moderately high specific surface area (lower than KB), enable the formation of extended conductive networks with more TPBs. Consequently, CNT-based SSCs exhibit more SE decomposition than SP-based SSCs but less than KB-based SSCs.

**Figure 2:**
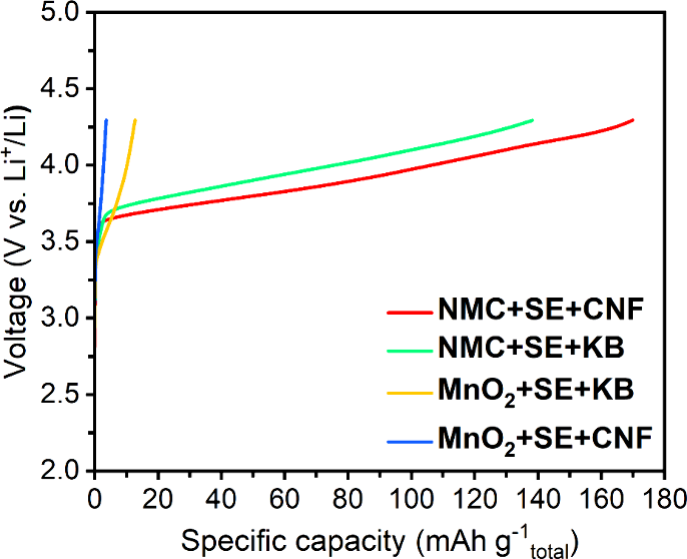
Comparison between solid-state cathodes with KB and CNFs on CAMs and MnO_2_, respectively. The load curves of the NMC+SE+CNF, NMC+SE+KB, MnO_2_+SE+KB, and MnO_2_+SE+CNF cathodes at 1 mA cm^–2^, 2 MPa stack pressure and 30 °C. The specific capacity is calculated based on the total mass of the solid-state cathode.

To investigate further the differences between SSCs with CNFs and KB, X-ray photoelectron spectra (XPS) were collected for each as a function of the state of charge (sulfur in Figure 3 and phosphorus in Figure S13). Considering Figure 3, the decomposition products are in accord with those observed previously for cells with carbon black.^16,20,63,64^ The pristine cells show the characteristic PS_4_^3–^ and S^2–^ peaks of Li_6_PS_5_Cl (see XPS for SE alone in Figure S14), with evidence of minor decomposition shown by the presence of a small amount of polysulfides and marginally more decomposition in the case of KB. Differences between the SSCs with CNFs and KB are seen on charging. In both cases, the quantities of decomposition products increase with state of charge. At a higher state of charge, elemental sulfur (−S^0^−) appears. However, the extent of decomposition is significantly greater in the case of KB than CNFs and the onset of decomposition to elemental sulfur occurs at a lower voltage, 3.8 V, in the case of KB compared with 4.3 V for CNFs. The corresponding compositional analysis is shown in the bar chart between the two sets of XPS data for CNFs and KB, respectively. Phosphorus XPS data in Figure S13 show very similar trends. They indicate the appearance of PO_*x*_ at 4.3 V for CNFs and 4 V for KB, and with more PO_*x*_ in the case of KB. This finding reinforces the sulfur XPS results and is also consistent with the time-of-flight secondary ion mass spectrometry (TOF-SIMS) data (Figure S15). These trends were further confirmed by collecting impedance data on cells with CNFs and KB, respectively (Figure S16). In the case of KB, a semicircle grows on cycling, which is consistent with a growing interphase at the triple phase boundary and is in accord with the SE decomposition. The semicircles significantly decrease when CNFs are used in the SSC, consistent with a significantly smaller amount of decomposition.

**Figure 3:**
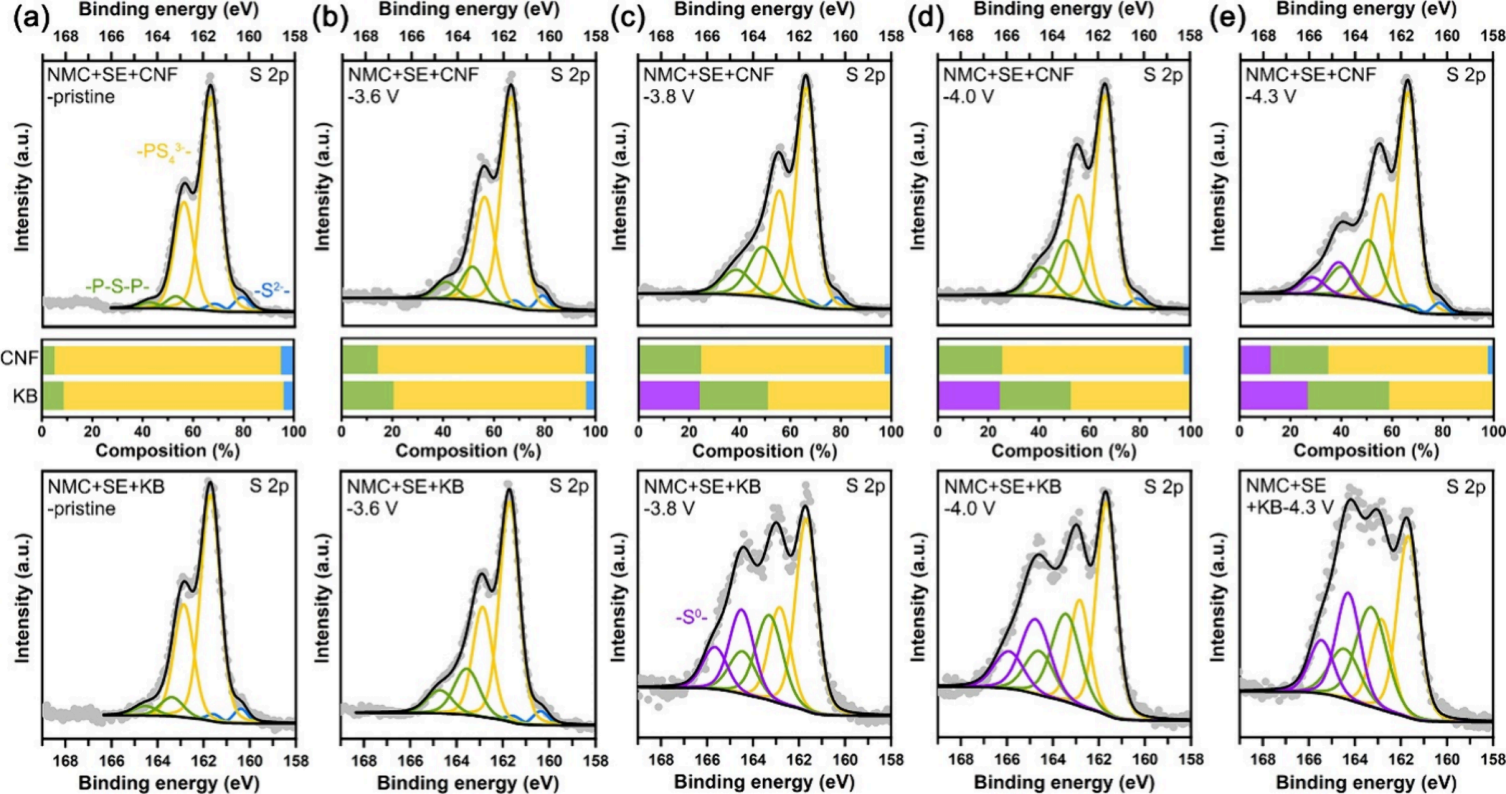
XPS analysis showing the different SE decomposition behaviors in the solid-state cathodes at different stages. S 2p XPS data and compositional analysis of the fitting results for the NMC+SE+CNF and NMC+SE+KB solid-state cathodes at the (a) pristine, (b) 3.6 V, (c) 3.8 V, (d) 4.0 V, and (e) 4.3 V charged states.

To explore how the CAM and carbon affect the decomposition individually in a practical SSC, we collected the XPS data shown in Figure 4 were collected. Figure 4a shows the results for CAMs and SEs alone without carbon charged to 4.3 V, whereas Figure 4b,c shows what happens when electrodes containing 3 wt % CNFs and KB respectively are charged to the same 4.3 V but with MnO_2_ instead of CAM. In all cases, decomposition only to polysulfides is observed and more predominantly for KB than CNFs in the electrodes with high CAM loading and ratio at practical cycling conditions. Crucially none of the results show decomposition to elemental −S^0^–. Elemental −S^0^– occurs only when carbon and CAMs are present together in a practical SSC. It should be noted that the interfacial side reactions are regulated by both the thermodynamics and dynamics. Therefore, the composition of final interfacial byproducts could be affected by many factors such as the CAM specific surface area, carbon specific surface area, CAM ratio, carbon ratio, SSC loading, cycling current density and cycling temperature, which explains the difference of final interfacial byproducts between our findings and previous results.^65,66^ However, what is clear is that the simultaneous presence of CAMs and carbon in the SSC induces more severe oxidative decomposition of the SE (*e.g*., form elemental −S^0^– in our case, Figure 3 and Figure 4), pointing to the importance of where the SE, CAM and carbon meet locally. In the case of KB, the nanometer-sized carbon particles appear at numerous locations where SSEs and CAMs meet, compared with CNFs. Reaction requires the transfer of ions/electrons across the interface, and the decomposition products accumulate across this new interphase layer. The presence of numerous carbon nanoparticles at the interface could facilitate electron transfer that in turn would increase the degree of decomposition as shown in Figure 5.

**Figure 4:**
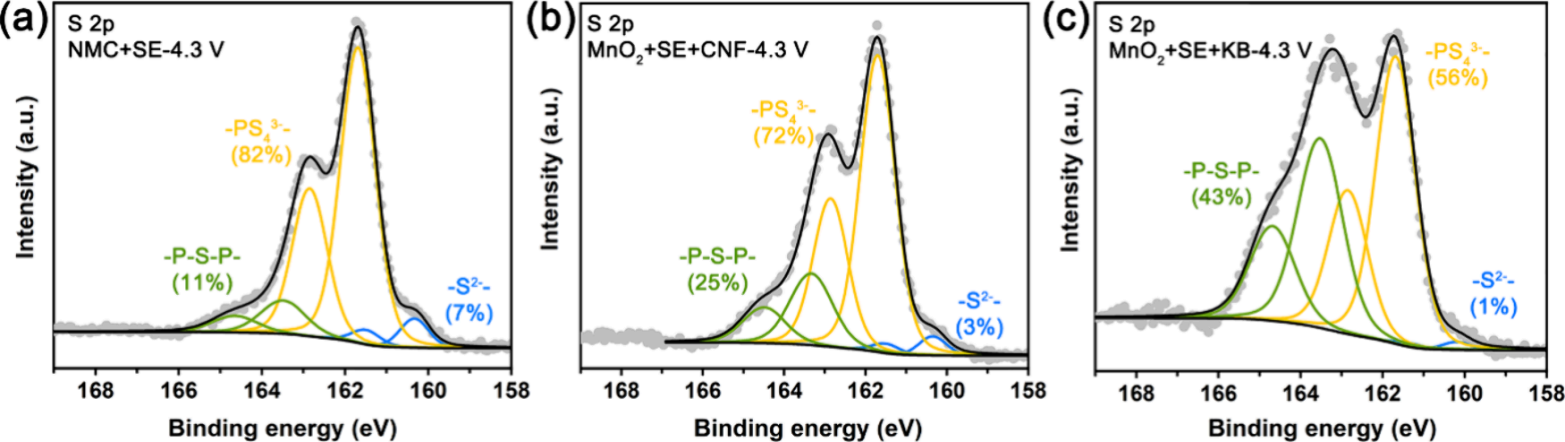
XPS analysis showing the effects of individual CAM or carbon on SE decomposition. S 2p XPS data of the (a) NMC+SE, (b) MnO_2_+SE+CNF, and (c) MnO_2_+SE+KB solid-state cathodes at the 4.3 V charged state.

**Figure 5:**
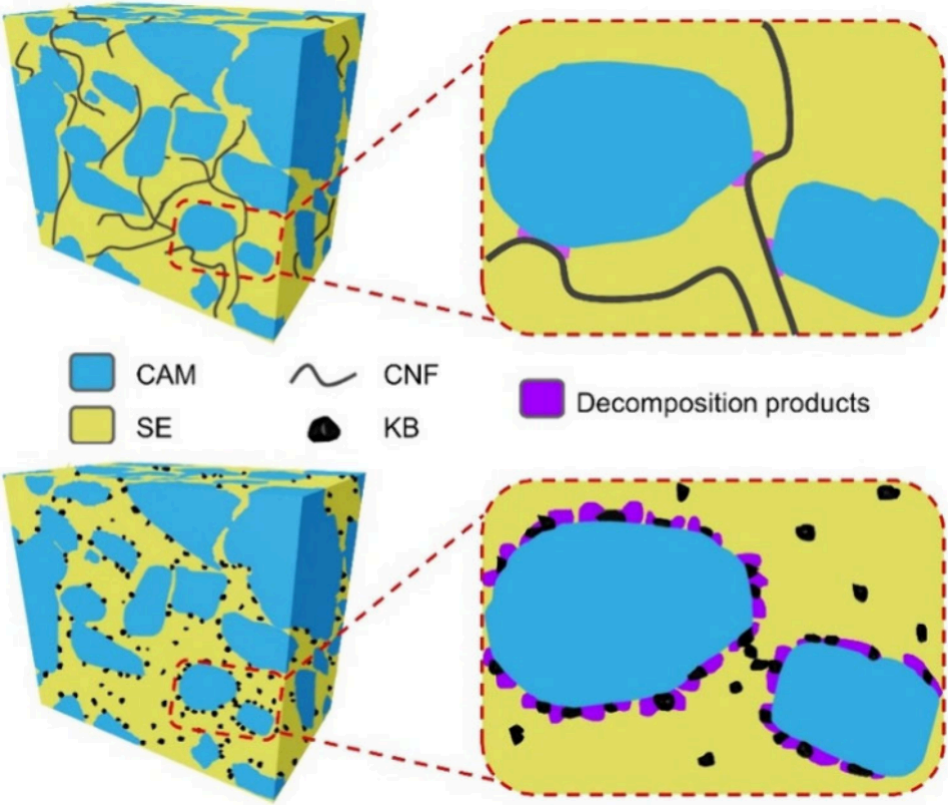
Schematics of the SSCs with CNFs and KB.

It is interesting to compare the rate capability of the SSC with CNFs with the SSC in a LIB based on the liquid electrolyte (Figure 6). The two cathodes have the same volume of electrolyte except that in one case the electrolyte is Li_6_PS_5_Cl and in the other LP30 (1 M LiPF_6_ in ethylene carbonate: dimethyl carbonate [1:1 v/v]). At low to intermediate rates, the capacity of the cathode is higher with the liquid electrolyte (Figure 6a), indicating that the ion transport through the composite electrode to the CAM particles is more effective due to the higher conductivity of the liquid electrolyte. However, as the rate increases, this difference decreases until at a current density of 8 mA cm^–2^, the rate capability of the SSC exceeds that of the cathode with the liquid electrolyte. At high rates, cells with a liquid electrolyte can suffer from concentration polarization, where on discharge, there is depletion of salt concentration near the CAM particles, limiting the rate capability.^67^ In contrast, ceramic electrolytes exhibit a transport number of 1 for the Li^+^ and there is no possibility of concentration polarization.^68^ The cycling efficiencies for the solid- and liquid-electrolyte-based cathodes at 1 mA cm^–2^ and 30 °C are shown in Figure 6b. In both cases, in the first cycle, the efficiencies are around 80%. The efficiencies then rise, however in the case of the SSC they remain above 99.5% whereas for LP30 the efficiency decreases continuously. These data are consistent with the continuous degradation of liquid electrolytes and the evolution of the cathode electrolyte interface layer, whereas the degradation of the SE in the SSC with a low specific surface area carbon slows significantly.^68^ Increasing the electrode thickness will cause a proportional increase in charge (electrons and ions) transport distance, tortuosity and fracture and delamination, all of which will result in more Li loss and thus lower Coulombic efficiency compared to the thin electrodes (≤5 mg cm^–2^) that were widely used in previously reports with LP30.^2,69–75^ Furthermore, without the electrolyte additives such as vinylene carbonate (VC), fluoroethylene carbonate (FEC), the cycle efficiency could be further decreased as the self-passivating cathode electrolyte interphase (CEI) cannot be formed in the pristine LP30 electrolyte.^7,76,77^ As a result, the low cycle efficiency with the pristine LP30 electrolyte is reasonable because we used an ∼110 μm thick electrode (∼25 mg cm^–2^) in this work.

**Figure 6:**
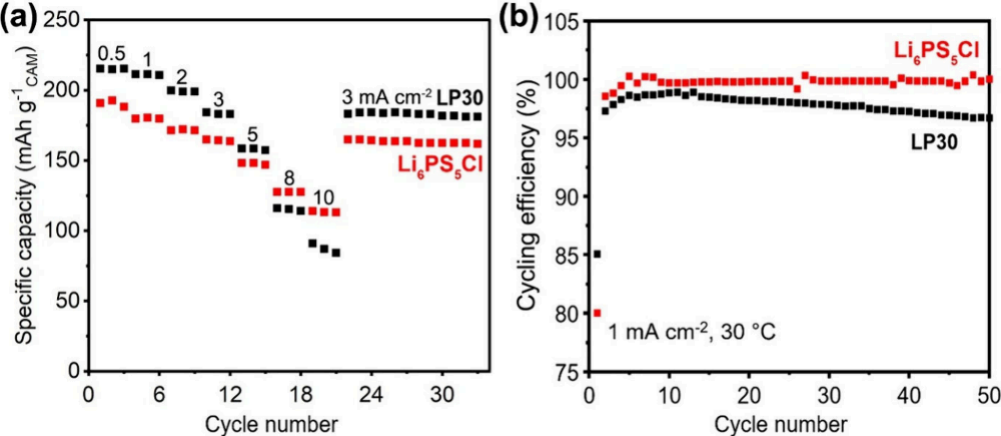
(a) Rate and (b) cycling efficiency comparison between cathodes with Li_6_PS_5_Cl and LP30 at 1 mA cm^–2^, 2 MPa stack pressure, and between 2.5 and 4.3 V at 30 °C.

Although the use of CNFs as the electronic additive in the SSC suppresses the decomposition of Li_6_PS_5_Cl largely to the first few cycles, the decomposition does restrict the performance.^78^ Raising the temperature of the cell with Li_6_PS_5_Cl to 80 °C results in a CAM utilization of 210 mAh g^–1^ even at a high current density of 3 mA cm^–2^ (Figure S17a), comparable to the cathode with a liquid electrolyte at 30 °C, indicating that at this temperature, mass transport through SEs and the interphase layer is no longer limiting. The cell can cycle at a much higher rate of 10 mA cm^–2^ (2 C) with utilization of the CAM at 185 mAh g^–1^ at the first cycle and 145 mAh g^–1^ after 500 cycles (Figure S17b).

The effect of double and triple phase boundaries on the SE decomposition is decoupled in the practical thick uncoated solid-state cathodes with a high CAM ratio (i.e., 75%) at practical cycling conditions. More severe oxidative decomposition of SEs only occurs when all three of the cathode active material, carbon, and the SE present simultaneously in the solid-state cathode, highlighting the importance of the triple phase boundary in decomposition and fading. The carbon nanoparticle with high specific surface area enables efficient electron transfer at the interface, increasing decomposition compared with carbon nanofibers, resulting in a lower capacity and faster fading. By regulating the electronic pathways at the triple phase boundary like using low specific surface area carbon nanofibers in solid-state cathodes, the decomposition reactions can be suppressed significantly, enabling an areal capacity of 4.6 mAh cm^–2^ (LiNi_0.83_Mn_0.06_Co_0.11_O_2_ utilization of 182 mAh g^–1^) and ∼85% capacity retention after 500 cycles at 30 °C, 1 mA cm^–2^, and 2 MPa between 2.5 and 4.3 V. Even without any coatings, higher LiNi_0.83_Mn_0.06_Co_0.11_O_2_ utilization and good cycling stability can be achieved via elevating the temperature. While the use of low surface area carbon nanofibers is likely beneficial generally in SSCs with other cathode active materials, such different active materials, even when combined with the same sulfide solid electrolyte used here, will result in different decomposition products and pathways.

## Supplementary Material



## Notes

Experimental methods, additional PXRD, SEM, FTIR and BET results for materials, FIB-SEM images, electronic conductivity, XPS and TOF-SIMS analysis of composite cathode, d*Q*/d*V* curve, EIS, load curves and cycling performance of all-solid-state batteries or liquid batteries (PDF)
