# Supplementary material for: Understanding the Role of Triple Phase Boundaries on Coating-Free Solid-State Cathodes
Source: ACS Energy Lett. 2026 Jan 30;11(2):1623–30. doi: 10.1021/acsenergylett.5c02607 (PMC12910715; doi:10.1021/acsenergylett.5c02607)
Supplement: Supplementary file 1 [file nz5c02607_si_001.pdf]

Supporting Information

Understanding The Role of Triple Phase Boundaries on Coating-Free Solid-State Cathodes

Longlong Wang,[1,2‡] *Bingkun Hu,*[2‡] *Christopher Doerrer,*[2‡] *Shengming Zhang,*[2] *Lechen Yang,*[2] *Liquan Pi,*[2] *Max Jenkins,*[2] *Boyang Liu,*[2] *Shengda D. Pu,*[2] *Yi Yuan,*[2] *Hui Gao,*[2] *Alex W. Robertson,*[2,3] *Patrick S. Grant,*[2] *Xiangwen Gao,*[1]* *Peter G. Bruce*[2,4]*

1. Future Battery Research Centre, Global Institute of Future Technology, Shanghai Jiao Tong University, Shanghai, 200240, China
2. Department of Materials, University of Oxford, Oxford, OX1 3PH, UK
3. Department of Physics, University of Warwick, Coventry, CV4 7AL, UK
4. Department of Chemistry, University of Oxford, Oxford, OX1 3QZ, UK

*Corresponding author.

Email address: xiangwen.gao@sjtu.edu.cn (Prof. X. W. Gao),

peter.bruce@materials.ox.ac.uk (Prof. P. G. Bruce)

‡These authors contributed equally to this work.

Experimental methods

Electrode preparation: All chemicals were dried under vacuum and stored in an Ar-filled glovebox (O_2 and H_2O levels < 1 ppm). For the solid-state cathodes (SSCs), uncoated single crystal $LiNi_{0.83}Mn_{0.06}Co_{0.11}O_2$ (MSE Supplies) or MnO_2 (Merke), ultra-fine Li_6PS_5Cl (MSE Supplies) and graphited carbon nanofibers (CNFs, Sigma-Aldrich), Ketjen black (KB, MSE Supplies), Super P (SP, Alfa Aesar) or carbon nanotubes (CNTs, Sigma-Aldrich) with a mass ratio of 75:22:3 were mixed in a planetary ball mill (Fritsch Pulverisette 7). For the $Li_4Ti_5O_{12}$-based anode, battery grade $Li_4Ti_5O_{12}$ (MTI), ultra-fine Li_6PS_5Cl and CNFs with a mass ratio of 45:47:8 were mixed in a planetary ball mill. The liquid electrolyte was LP30 ($LiPF_6$ in ethylene carbonate: dimethyl carbonate (1:1 v/v), BASF battery-grade). The SSCs for liquid cells were composed of single crystal $LiNi_{0.83}Mn_{0.06}Co_{0.11}O_2$, carbon black and polyvinylidene fluoride (PVDF) with a mass ratio of 94:4:2 (the same volume for the liquid electrolyte as the SSCs) and the thickness was controlled to be the same at 110 μm.

Cell assembly: Solid-state cells were assembled in a custom-designed module with a PEEK mold and stainless steel plungers.[1–3] The inner diameter is 5 mm. A three-electrode cell was used in this work with Li metal as the reference. A series of layers comprising the SSC mixture (corresponding to an areal capacity of ~5 mAh cm^{-2}), 20 mg of Li_6PS_5Cl and ~80 mg cm^{-2} of the LTO-based anode mixture were pressed at 400 MPa for 5 mins. An external stack pressure of 2 MPa was used when making measurements on the solid-state cells. All work was carried out in an Ar-filled glovebox with O_2 and H_2O level below 1 ppm.

Electrochemical measurements: Galvanostatic cycling were performed using a VMP3 electrochemical workstation (Biologic) and a battery test system (CT-4008T, Neware). For the impedance analysis during cycling, the electrochemical impedance spectroscopy (EIS) was carried out after each cycle, using a Gamry Instruments Interface-1000 potentialstat with a

voltage amplitude of 10 mV in the frequency range from 1 MHz to 1 Hz. For the conductivity measurement, the solid electrolyte or solid-state cathode powder was pressed at 400 MPa in the polyether ether ketone (PEEK) mold to make the pellet. Two carbon papers were used as the blocking electrodes. Electrochemical impedance spectroscopy (EIS) and direct current (DC) polarization measurements were performed to determine the ionic and electronic conductivities, respectively, under an external pressure of 2 MPa in an Ar-filled glovebox.

Characterization: Power X-ray diffraction (PXRD) measurements were carried out in a Rigaku MiniFlex inside the glovebox. Scanning electron microscopy (SEM) images were taken using a Zeiss-Merlin microscope with an airtight transfer holder avoiding exposure to air. Plasma focused ion beam scanning electron microscopy (PFIB-SEM) was used to characterize the cross-section morphology of the electrode. Brunauer-Emmett-Teller (BET) specific surface area analysis was carried out using a Micromeritics 3Flex Adsorption Analyser. Samples were dried under vacuum at 70 °C overnight prior to analysis and further dried with in situ degassing at 70 °C under high vacuum for 4 hours once assembled on the analyser. Attenuated total reflectance-Fourier transform infrared (ATR-FTIR) spectra were acquired with a Thermo Fisher Nicolet iS50 spectrometer in a N_2-filled glovebox. Time-of-flight secondary ion mass spectrometry (TOF-SIMS) was performed with a TOF.SIMS 5 instrument (IONTOF GmbH, Germany). The primary and secondary ion guns used were 25 keV Bi^+ and 10 keV gas cluster Ar^+ (GCIB-Ar), respectively. Samples were transferred under an argon atmosphere, and the sample surface was cleaned with 1 keV Cs^+ sputter gun for 2 min to eliminate contamination. All TOF-SIMS measurements were performed in negative ion mode to probe the chemical nature of the degradation products. X-ray photoelectron spectroscopy (XPS) measurements were conducted using a PHI5000 Versa Probe III instrument (Ulvac-PHI, Inc., Chigasaki, Japan). An Al monochromatic source was used to generate X-rays with a power of 25 W, a voltage of 15 kV and a beam diameter of 100 microns. A pass energy of 55 eV was set for the

analyzer. An electron neutralizer gun was used to prevent any surface charge build-up. The analysis and fitting were carried out using the CasaXPS software package.

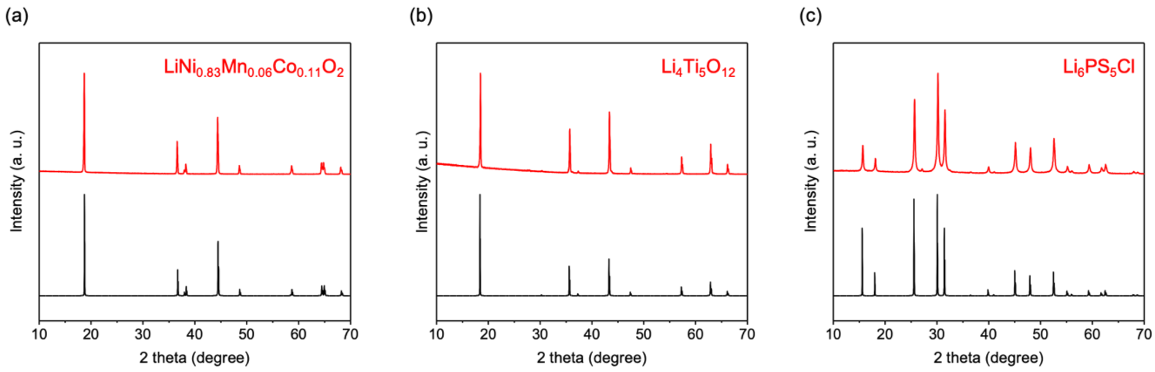

Figure S1. PXRD patterns of (a) $LiNi_{0.83}Mn_{0.06}Co_{0.11}O_2$, (b) $Li_4Ti_5O_{12}$ and (c) Li_6PS_5Cl and the standard patterns (black).

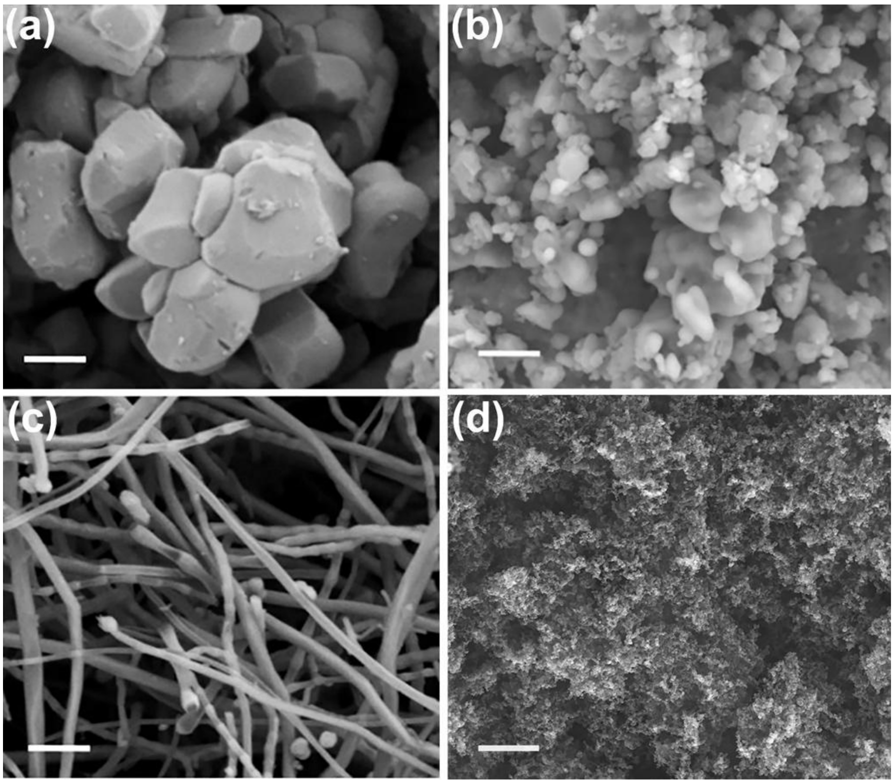

Figure S2. SEM images of (a) $LiNi_{0.83}Mn_{0.06}Co_{0.11}O_2$, (b) Li_6PS_5Cl, (c) CNFs and (d) KB. The scale bar is 1 µm.

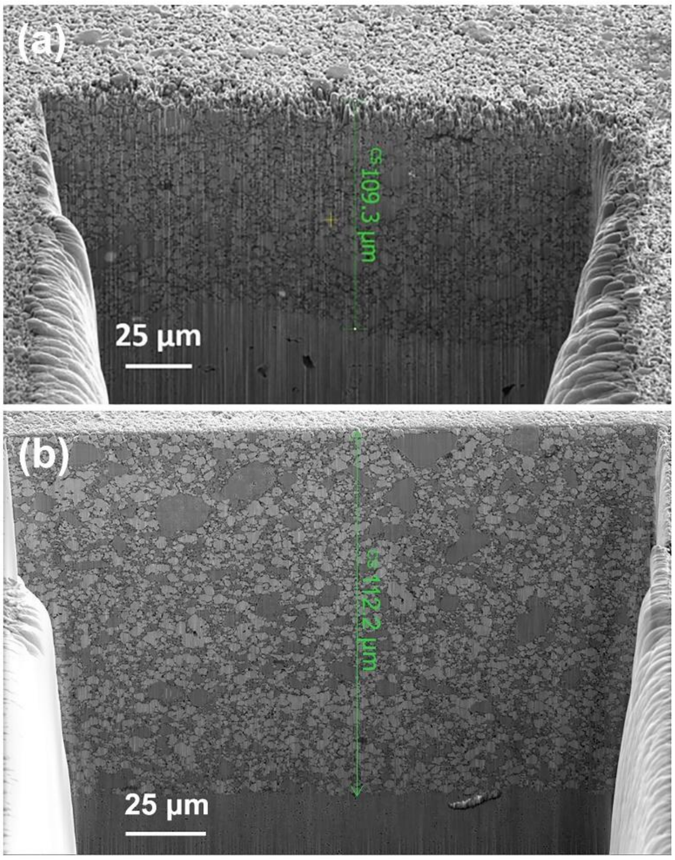

Figure S3. The cross-sectional FIB-SEM image of the composite cathode with (a) CNFs and (b) KB.

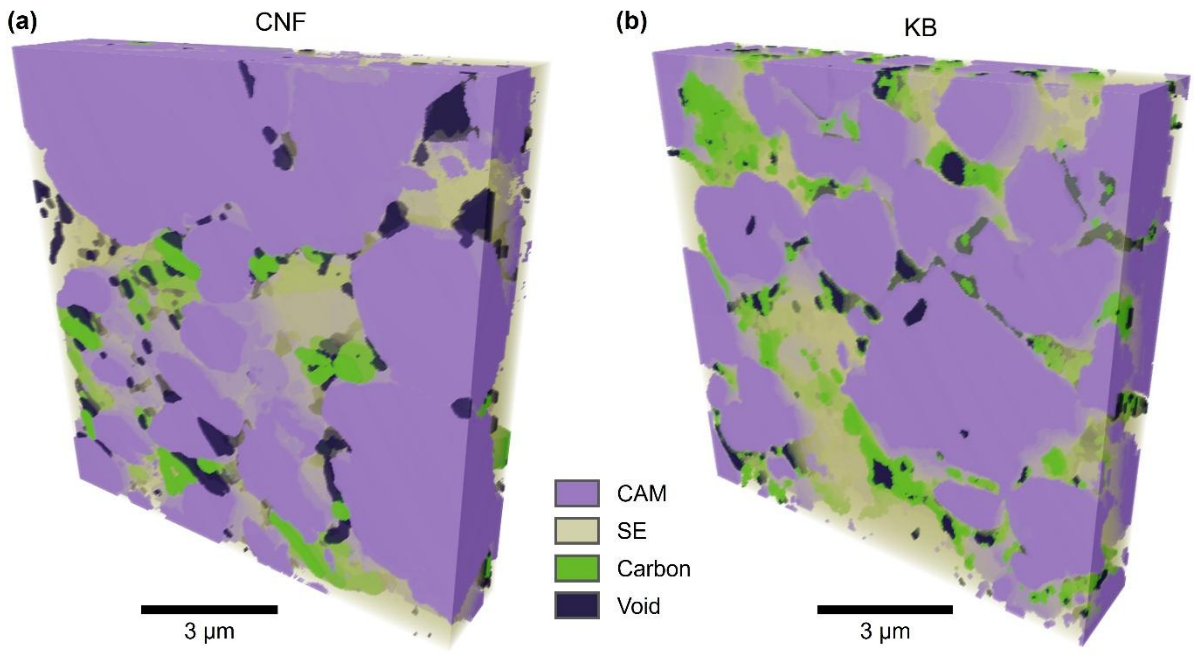

Figure S4. Plasma focused ion beam scanning electron microscopy (PFIB-SEM) reconstructed 3D morphology of the composite cathode with (a) CNFs or (b) KB. The obtained volumetric ratios of CAM: SE: carbon for CNFs and KB are both around 49:42:5 (4% void). The reconstructed volume is 10 μm × 10 μm × 2.5 μm.

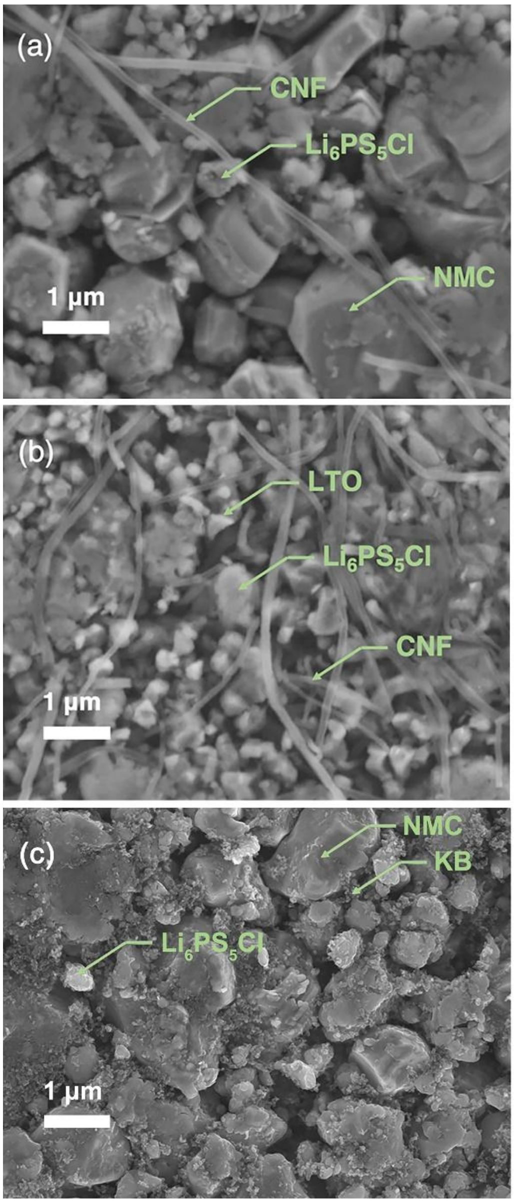

Figure S5. SEM images of (a) the NMC+Li_6PS_5Cl+CNF solid-state cathode, (b) LTO+Li_6PS_5Cl+CNF solid-state anode and (c) NMC+Li_6PS_5Cl+KB solid-state cathode.

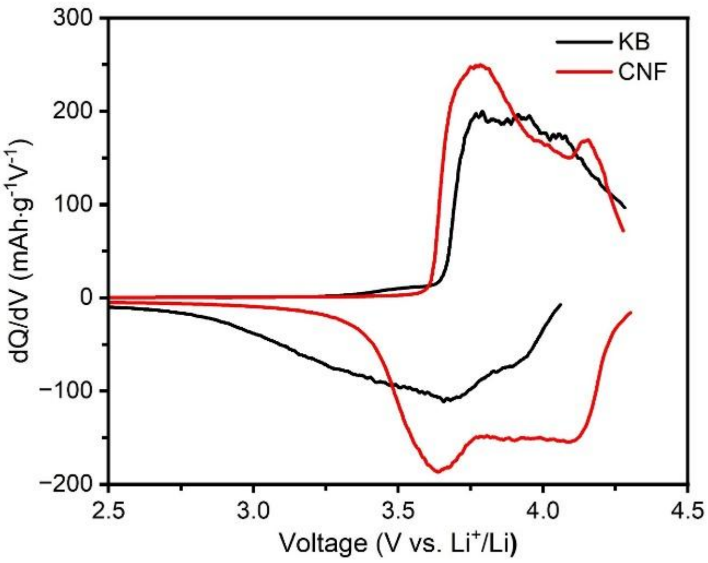

Figure S6. Differential capacity vs. voltage (dQ/dV) curves for CNF- and KB-based solid-state cathodes in the first cycle at 1 mA cm^{-2}, 2 MPa stack pressure and 30 °C.

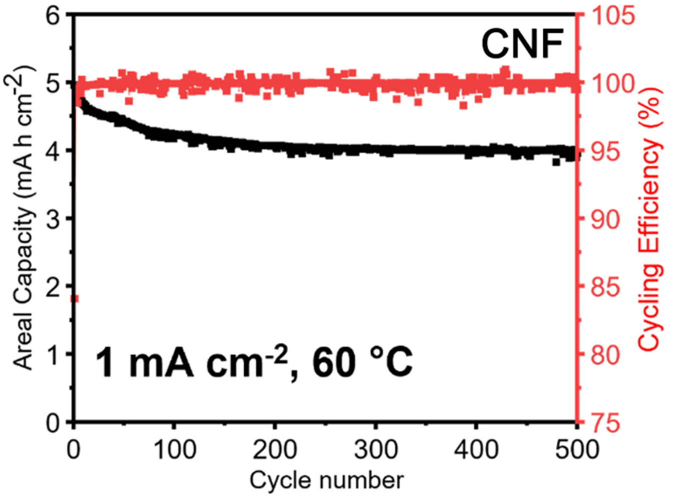

Figure S7. The capacity and cycling efficiency of the CNF-based solid-state cathode over 500 cycles at 1 mA cm^{-2}, 2 MPa stack pressure and 60 °C.

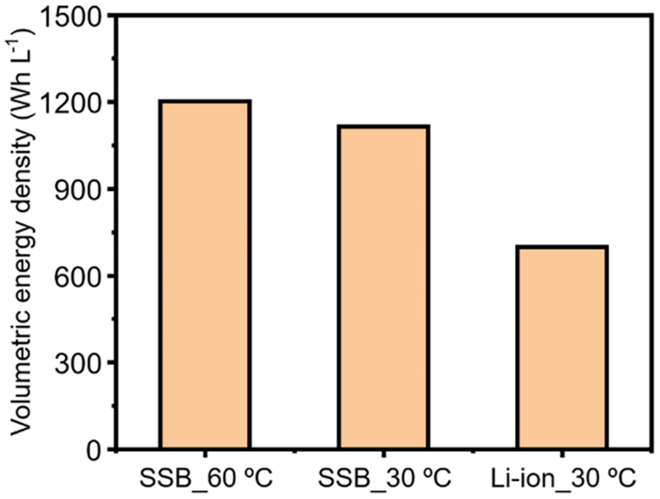

Figure S8. The volumetric energy density comparison between a Li-ion cell of today and solid-state cells with this CNF-based solid-state cathode, a 20 μm Li-metal anode and a 20 μm Li_6PS_5Cl separator.

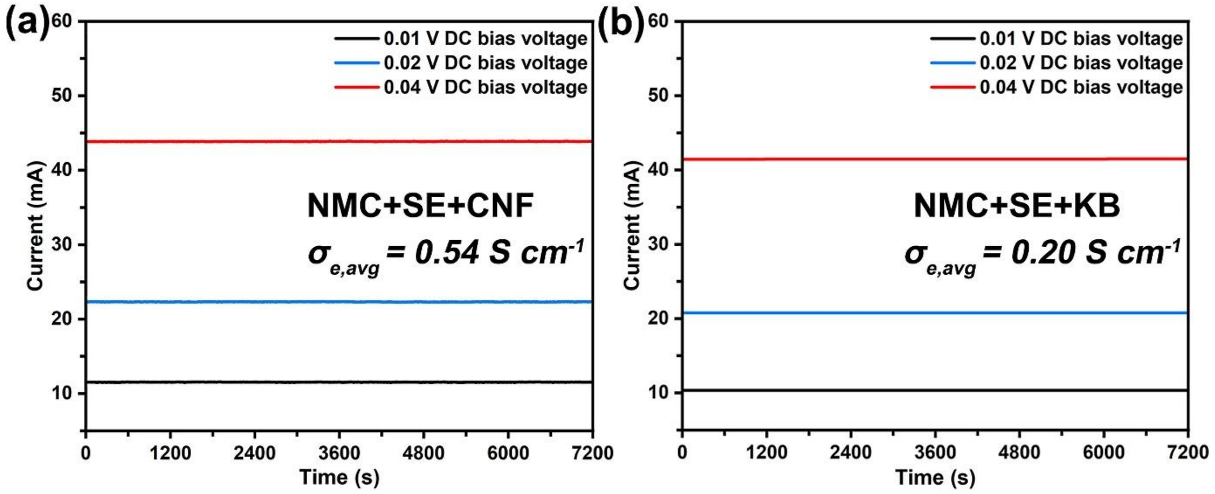

Figure S9. Electronic conductivity of the NMC+SE+CNF and NMC+SE+KB based solid-state cathodes. Current–time curve of (a) the cell consisting of carbon paper/NMC+SE+CNF solid-state cathode/carbon paper and (b) the cell consisting of carbon paper/NMC+SE+KB solid-state cathode/carbon paper at 2 MPa stack pressure and 30 °C.

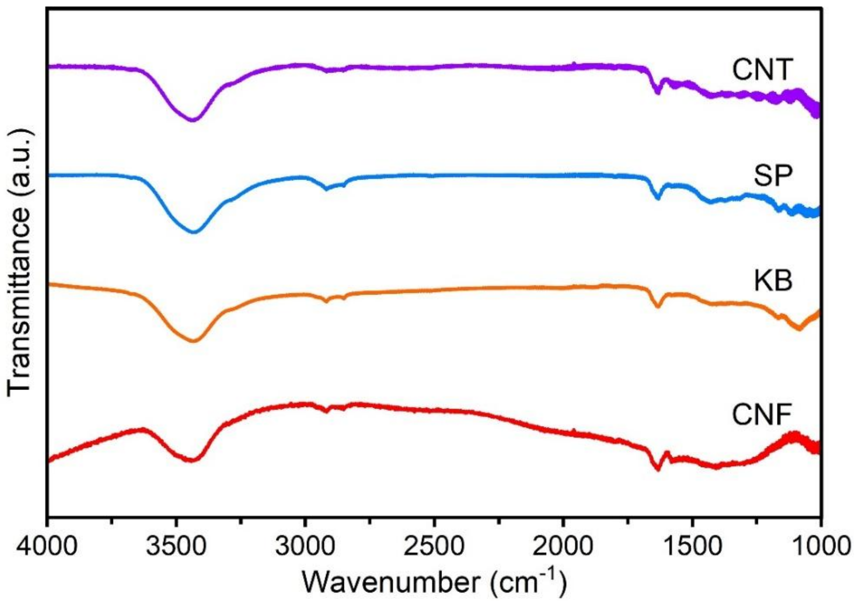

Figure S10. FTIR spectra of the dried carbon nanofibers (CNFs), Ketjen black (KB), Super P (SP) and carbon nanotubes (CNTs).

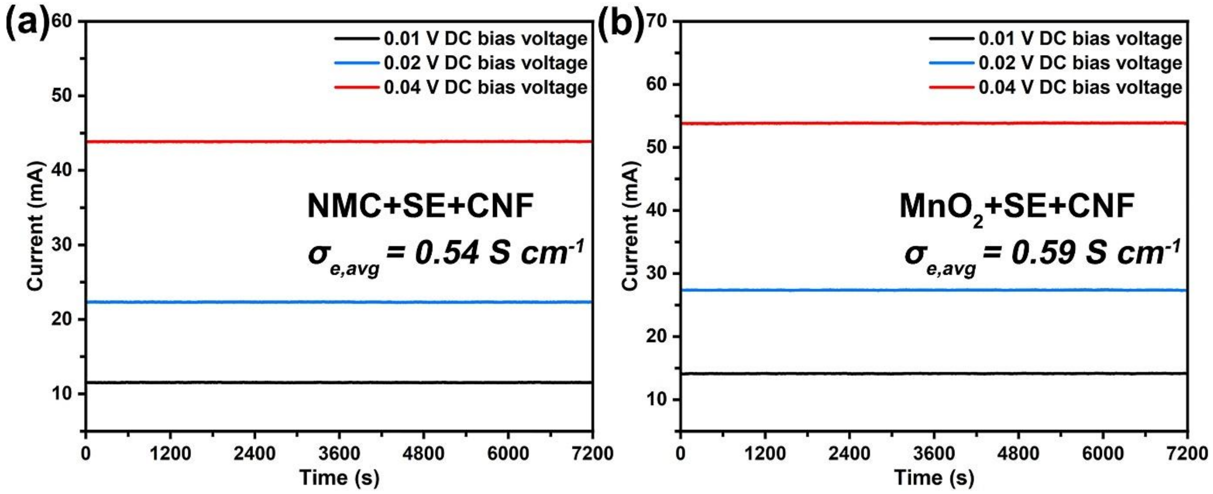

Figure S11. Electronic conductivity of the NMC+SE+CNF and MnO_2+SE+CNF based solid-state cathodes. Current–time curve of (a) the cell consisting of carbon paper/NMC+SE+CNF solid-state cathode/carbon paper and (b) the cell consisting of carbon paper/MnO_2+SE+CNF solid-state cathode/carbon paper at 2 MPa stack pressure and 30 °C.

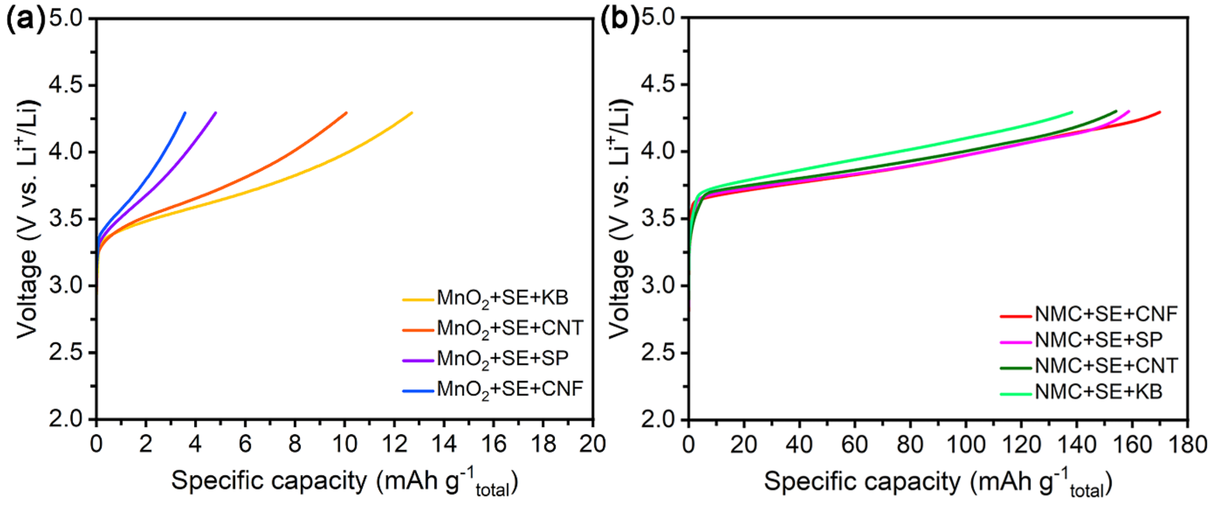

Figure S12. Comparison between solid-state cathodes with carbon nanofibers (CNFs), Super P (SP), carbon nanotubes (CNTs), and Ketjen black (KB) on NMC and MnO_2 respectively at at 1 mA cm^{-2}, 2 MPa stack pressure and 30 °C. (a) The load curves of the MnO_2+SE+KB, MnO_2+SE+CNT, MnO_2+SE+SP and MnO_2+SE+CNF cathodes. (b) The load curves of the NMC+SE+CNF, NMC+SE+SP, NMC+SE+CNT and NMC+SE+KB cathodes. The specific capacity is calculated based on the total mass of the solid-state cathode.

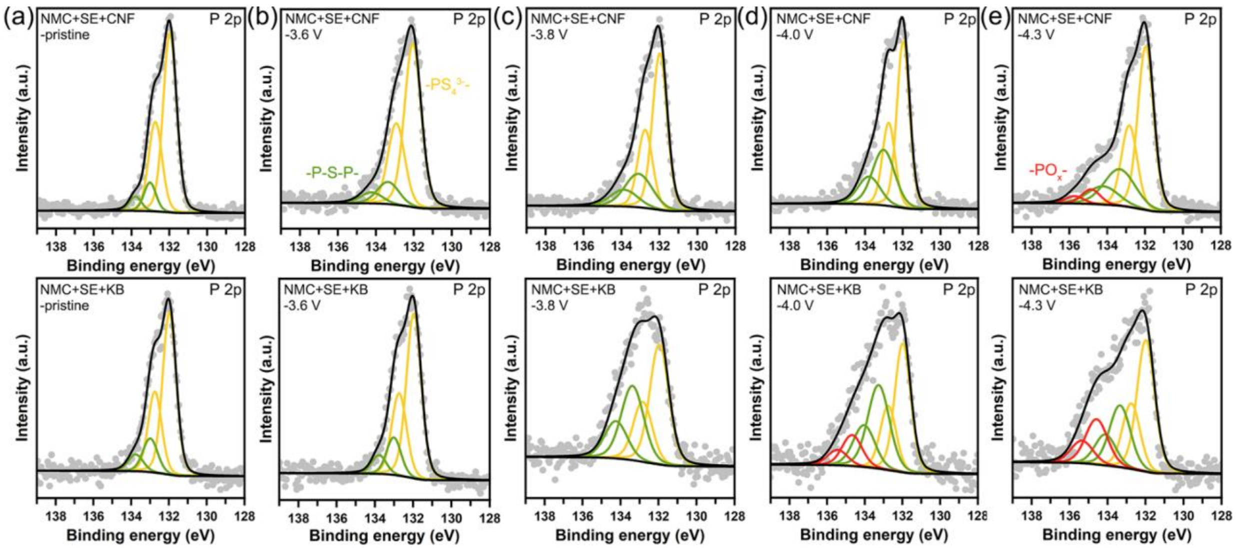

Figure S13. XPS analysis showing the different SE decomposition behaviour in the solid-state cathodes at different stages. P 2p XPS data of the fitting results for the NMC+SE+CNF and NMC+SE+KB solid-state cathodes at the pristine (a), 3.6 V (b), 3.8 V (c), 4.0 V (d) and 4.3 V (e) charged states.

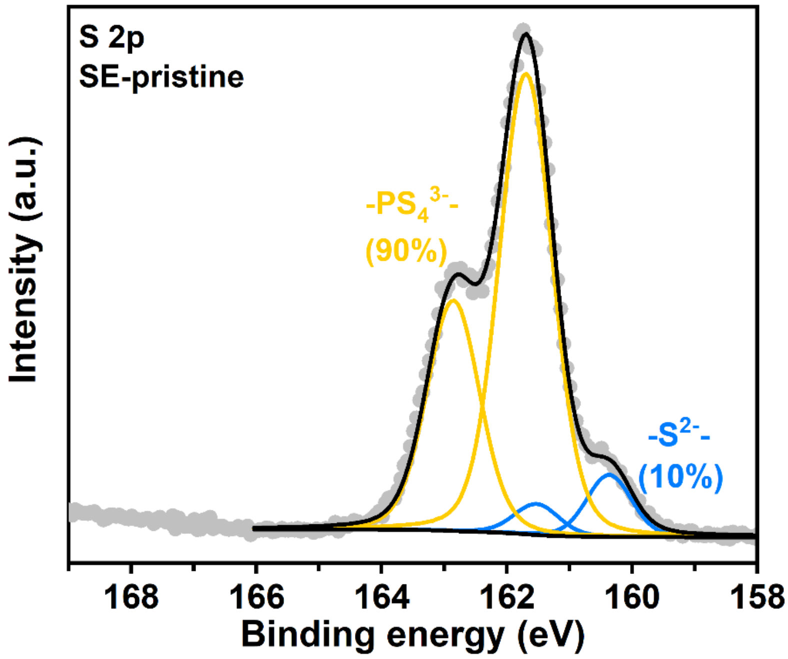

Figure S14. S 2p XPS data of the pristine Li_6PS_5Cl.

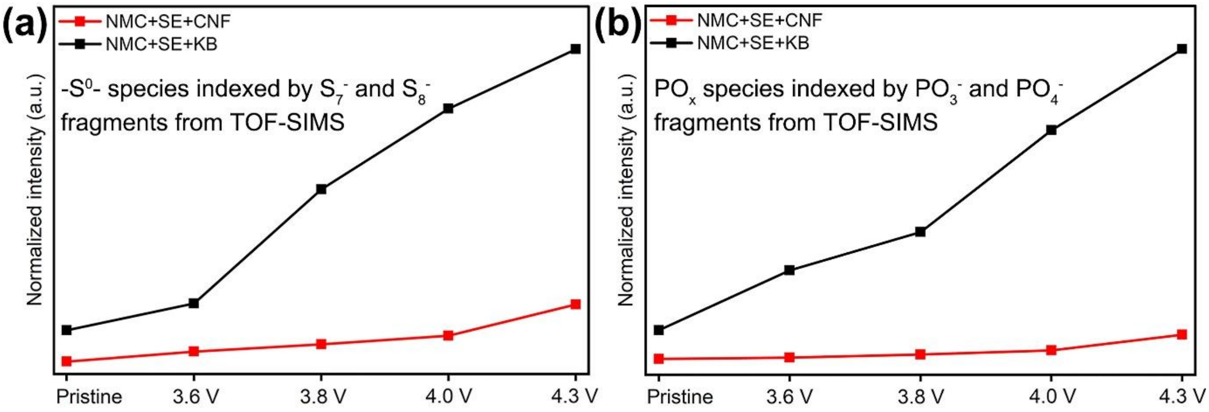

Figure S15. TOF-SIMS investigation of -S^0- and PO_x species in the NMC+SE+CNF and NMC+SE+KB solid-state cathodes at the pristine, 3.6 V, 3.8 V, 4.0 V and 4.3 V charged states. (a) Evolution of -S^0- species indexed by S_7^- and S_8^- fragments. (b) Evolution of PO_x species indexed by PO_3^- and PO_4^- fragments.

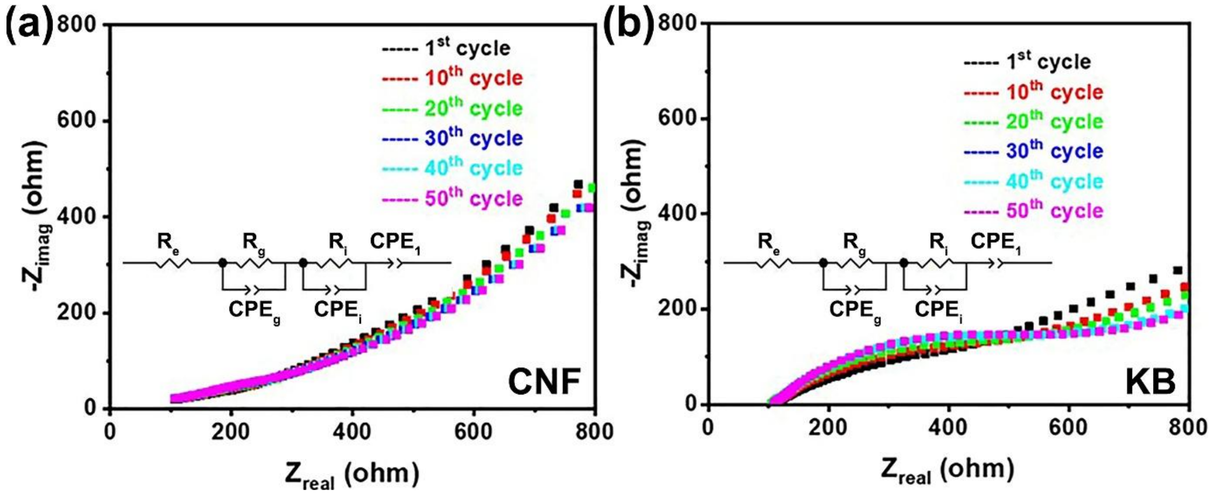

Figure S16. The Nyquist plots of the solid-state cathode with CNF (a) and KB (b) over 50 cycles, showing growth of semicircle on cycling on cathodes with high surface KB, whereas the semicircle is much smaller when CNF is substituted for KB. The suggested (logical) equivalent circuit is shown in the inset, where R_e, R_g-CPE_g, R_i-CPE_i and CEP_1 denote the electrolyte bulk resistance, electrolyte grain-boundary resistance, interfacial resistance from both the cathode and anode interfaces and Warburg resistance inside the cathode, respectively.

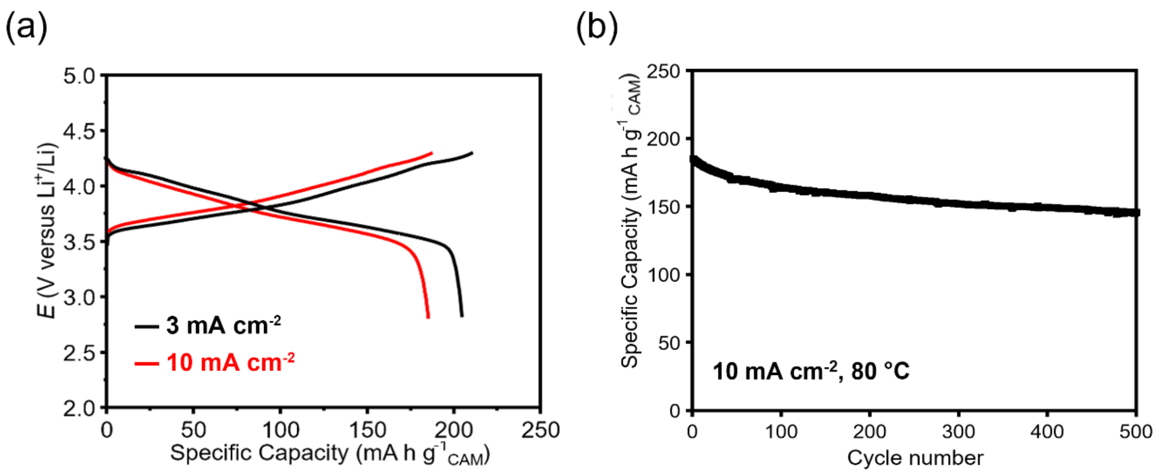

Figure S17. The electrochemical performance of the solid-state cathode at 80 °C. (a) The load curves at 3 mA cm^{-2} and 10 mA cm^{-2} (2 C). (b) the cycling behavior of the cathode at 10 mA cm^{-2} (2 C). The stack pressure is 2 MPa.

Table S1. Calculation basis for the volumetric energy density of solid-state cells with our CNF-based solid-state cathode.

Our cathode	CAM capacity (mAh g^{-1})	182 (30 °C)	197 (60 °C)
	CAM ratio (%)	75	
	Thickness (μm)	110	
	Areal capacity (mAh cm^{-2})	4.583	4.974
SSE	Assumed thickness (μm)	20	
Li-metal anode	Assumed thickness (μm)	20	
Area (cm^2)		0.19625	
Total thickness (μm)		150	
Discharge V_{ave} (V)		3.65	
Cell capacity (Ah)		8.9941×10^{-4}	9.7615×10^{-4}
Energy density (Wh L^{-1})		1115	1210

Table S2. The surface area of the CAM and two carbons in the SSC.

Material	BET surface area (m^2 g^{-1})
CNF	24 ± 1
KB	1010 ± 4
NMC	4 ± 0.1

References

(1) Doerrer, C.; Capone, I.; Narayanan, S.; Liu, J.; Grovenor, C. R. M.; Pasta, M.; Grant, P. S. High energy density single-crystal NMC/Li_6PS_5Cl cathodes for all-solid-state lithium-metal batteries. *ACS Appl. Mater. Interfaces* **2021,** *13* (31), 37809−37815.

(2) Doerrer, C.; Gao, X.; Bu, J.; Wheeler, S.; Pasta, M.; Bruce, P. G.; Grant, P. S. Fast-charging all-solid-state battery cathodes with long cycle life. *Nano Energy* **2025,** *134*, 110531.

(3) Wang, L.; Mukherjee, A.; Kuo, C. Y.; Chakrabarty, S.; Yemini, R.; Dameron, A. A.; DuMont, J. W.; Akella, S. H.; Saha, A.; Taragin, S.; Aviv, H.; Naveh, D.; Sharon, D.; Chan, T. S.; Lin, H. J.; Lee, J. F.; Chen, C. T.; Liu, B.; Gao, X.; Basu, S.; Hu, Z.; Aurbach, D.; Bruce, P. G.; Noked, M. High-energy all-solid-state lithium batteries enabled by Co-free $LiNiO_2$ cathodes with robust outside-in structures. *Nat. Nanotechnol.* **2024,** *19* (2), 208−218.
